# COVID-19 Infection among Family and Friends: The Psychological Impact on Non-Infected Persons

**DOI:** 10.3390/brainsci12091123

**Published:** 2022-08-24

**Authors:** Jagdish Khubchandani, Sushil Sharma, Fern J. Webb, Michael J. Wiblishauser, Manoj Sharma

**Affiliations:** 1 College of Health, Education & Social Transformation, New Mexico State University, Las Cruces, NM 88003, USA; 2 Provost Office, Texas A&M University Texarkana, Texarkana, TX 75503, USA; 3 College of Medicine, University of Florida, Jacksonville, FL 32209, USA; 4 School of Education, Health Professions & Human Development, University of Houston-Victoria, Victoria, TX 77901, USA; 5 School of Public Health, University of Nevada, Las Vegas, NV 89119, USA

**Keywords:** COVID-19, pandemic, infection, depression, anxiety, stress, psychiatry

## Abstract

Little is known about the mental health impact of having a family member or friend infected with COVID-19. Thus, the purpose of this study was to conduct a comprehensive national assessment of the psychological impact of COVID-19 infection, hospitalization, or death among family members and friends. A multi-item valid and reliable questionnaire was deployed online to recruit adults in the U.S. A total of 2797 adult Americans without a history of COVID-19 infection participated in the study and reported that they had a family member or friend infected with (54%), hospitalized due to (48%), or die (36%) of COVID-19 infection. Symptoms of depression, anxiety, or both (i.e., psychological distress) were statistically significantly higher among those who had family members/friends infected, hospitalized, or die due to COVID-19. Also, this study found that the greater the number of family members/friends affected by COVID-19, or the more severe the COVID-19 infection outcome (i.e., hospitalization vs. death), the higher the odds of symptoms of depression, anxiety, or both. There is an urgent need to develop educational interventions and implement policy measures that address the growing mental health needs of this subgroup of the population that was not infected but indirectly affected by COVID-19 infections among social networks.

## 1. Introduction

The COVID-19 pandemic has caused major upheavals for societies and individuals. By July 2022, more than six million individuals died of COVID-19 infections and more than 550 million were infected worldwide [1]. Beyond the direct health impact of COVID-19 via infections, hospitalizations, and deaths, many other population health problems and risky health behaviors have been extensively explored and reported in the literature during the pandemic [2,3,4,5,6,7]. Among the most prominent public health issues explored during the COVID-19 pandemic, mental health problems have been reported extensively across regions and populations. For example, a global review from the early stages of the pandemic found that the prevalence of depression, anxiety, and distress symptoms worldwide were 28.18%, 29.57%, and 25.18%, respectively. In contrast, another review of data from the first year of the pandemic suggested that the prevalence of depression, anxiety, and distress was 31.4%, 31.9%, and 41.1%, respectively, indicating sharp increases [8,9]. Several reviews also focused on special populations. For instance, a review of healthcare workers (HCWs) with data till April 2020 found the prevalence of depression and anxiety to be 24.1% and 28.6%, respectively. In contrast, another review of HCWs with data till November 2020 found the prevalence of anxiety and depression to be 40% and 37%, respectively [10,11]. Across all these studies and reviews from various regions and populations, it was noted that the rates of psychological problems among individuals worldwide increased substantially during the pandemic [7,8,9,10,11].

A plethora of reasons have been cited for the increase in psychological distress and poor mental health during the COVID-19 pandemic. These findings could be attributed to a variety of factors such as personality type and pre-pandemic mental health status, level of control over life, working conditions, household wealth, community resources, social support, COVID-19-related fears and worries, and above all, resilience [12,13,14,15]. For example, an investigation with more than 15,000 European adults earlier in the pandemic explored resilience and found that good stress response and positive appraisal, specifically of the consequences of the COVID-19 crisis, were strong predictors of good mental health. For others, however, since the beginning of the pandemic, stress and associated maladaptive coping resulted in various health risk behaviors and problems. Subsequently, within the first year of the COVID-19 pandemic [13,14,15], prolonged lockdowns and social distancing, isolation and boredom, job insecurity and financial problems, uncertainty related to the future, and worries and fear were the most common factors cited for poor mental health [6,7,8,9,12,13,14]. More recently, misinformation and rumors, internet addiction or excessive social and mass media consumption, work-life imbalance or altered family responsibilities, occupational losses or changes, food insecurity and material deprivation, the stigma of infection or becoming infected with COVID-19, fear of COVID-19 infection, and prevailing social and political upheaval have also been explored with regards to mental health during the pandemic [14,15,16,17,18,19].

Despite the extant literature on psychological distress during the pandemic, little is known about the psychological impact of COVID-19 infections among family and friends. Thus, the purpose of this investigation was to assess the psychological health of a national random sample of adult Americans who were not infected with COVID-19 but had family members or friends infected with COVID-19 virus. Specifically, we aimed to investigate the proportion of individuals who had a family member or friend infected/hospitalized/die due to COVID-19, the prevalence of symptoms of depression and anxiety, and the differences in the burden of anxiety and depression symptoms based on whether or not someone had a family member or friend infected/hospitalized/die due to COVID-19.

## 2. Methods

A multi-component valid and reliable questionnaire was deployed via Amazon mTurk and emails to social networks and community organizations across the United States from June-September 2021 [12,13,15,17]. To estimate the required sample size, an a priori power analysis was conducted. Based on the total population of adults in the USA (*n* ≈ 250 million), 99% confidence levels, and a conservative 3% margin of error, a total of 1844 participants were needed for the study (assuming that half of them knew someone who was infected, hospitalized, or died of COVID-19). A total of 2797 adult Americans participated in this study (exceeding the required sample size) [12,15]. A comprehensive literature review was conducted to compile a draft survey with face validity. This initial survey draft was reviewed by a panel of experts (*n* = 3) to ensure content validity. Based on the feedback of the experts, several changes were made to the draft to create the final questionnaire [15,16,17,18,19,20]. The final survey consisted of three major sections (i.e., questions on COVID-19 infection, psychological distress, and sociodemographic information of the study participants). To screen for potential participants, we asked the study participants if they had ever tested positive for COVID-19 infection (with response options ‘yes’ vs. ‘no’). Only those who never tested positive for COVID-19 infection or never had COVID-19 infection were included in this study.

The study participants were asked if they knew someone (i.e., family members or friends) who tested positive for COVID-19, was hospitalized due to COVID-19 or died due to COVID-19 infection. The response options for these questions were: ‘*yes, one person’*; ‘*yes, more than one person*’; or ‘*no, I do not know anyone*’ (infected with, hospitalized for, died due to COVID-19). The internal consistency reliability was computed for these three COVID-19 infection-related questions and was found to be high (Cronbach alpha = 0.86). Next, using the reliable and valid Patient Health Questionnaire-4 (PHQ-4) we assessed symptoms of anxiety (GAD-2), depression (PHQ-2), or both (PHQ-4) in the study sample [17,18,19,20]. Internal consistency reliability for the PHQ-4 components was computed and found to be reasonable (GAD-2 alpha = 0.74; PHQ-2 alpha = 0.72; PHQ-4 alpha = 0.83). In the last section of the survey, study participants were asked about their sociodemographic characteristics. IRB approval was sought before data collection for the study.

Data were analyzed using IBM SPSS 26 software. In the primary approach, we computed descriptive statistics (i.e., frequencies and percentages) to delineate the sociodemographic characteristics of the study population and the distribution of COVID-19 infection, hospitalizations, and deaths in the social networks of the study participants. Subsequently, Chi-square tests were conducted to assess group differences in symptoms of anxiety, depression, or both (psychological distress) based on sociodemographic characteristics and COVID-19 infection among friends and family members. Finally, a multiple regression analysis was conducted to explore the relationship between COVID-19 infection among family members/friends and psychological distress. Depression, anxiety, or symptoms of both, were the outcome variables while COVID-19 among family members/friends was treated as the predictor variables (where the ‘no’ infection, hospitalization, or death groups were used as comparison groups). Adjusted odds ratios (AOR with 95% confidence intervals) were computed for the probability of psychological distress based on COVID-19 among family/friends. Statistical significance was set at *p* < 0.05.

## 3. Results

A total of 2797 adult Americans without COVID-19 infection history participated in the study where the majority (>50%) were male, 18–35 years old, married, with children at home, White, employed full-time, college graduates, with incomes ≤ $60,000, and urban dwellers (Table 1). With regards to the psychological outcomes, almost a third or more of the study participants reported anxiety symptoms (33%), depression symptoms (43%), or symptoms of both anxiety and depression (psychological distress; 32%) (Table 1). These symptoms differed statistically significantly by demographic characteristics and the prevalence of these symptoms was highest for 18–35-year-olds, married individuals, those with children at home, college degree holders, those with incomes ≤ $60,000, or living in rural areas. White individuals were most likely to have anxiety symptoms (38%) while Hispanics were more likely to have depression symptoms (54%). Those working full-time were more likely to have anxiety symptoms (34%) while part-time employed persons were more likely to have depression symptoms (45%) (Table 1).

When asked about COVID-19 infection among family members and friends, 16% knew one person and 38% knew more than one COVID-19 infected person (Table 2). Similarly, 20% knew one person and 28% knew more than one person hospitalized due to COVID-19 infection. More than a third (36%) of participants had at least one family member or friend die due to COVID-19 infection. Individuals who had a family member/friend infected, hospitalized, or die due to COVID-19 infection had statistically significantly higher rates of anxiety, depression, or symptoms of both (Table 2). Compared to those who did not have a family member/friend affected by COVID-19 infections, knowing even one COVID-19 affected person increased the rate of psychological distress, and having more than one family member/friend affected by COVID-19 infection further increased the rate of psychological distress among study participants (Table 2).

Multiple logistic regression analyses were conducted to assess the association between psychological distress and having a family member/friend who was infected, hospitalized, or die due to COVID-19 infection (Table 3). Those who did not know anyone who was infected, hospitalized, or die due to COVID-19 were the comparison group in the logistic regression. Despite adjustment for sociodemographic characteristics, individuals who reported having family members/friends infected, hospitalized, or die due to COVID-19 were statistically significantly more likely to report psychological distress. For anxiety symptoms, knowing one infected person [AOR = 1.70 (95% CI = 1.34–2.15)] or more than one infected person [AOR = 2.14 (95% CI = 1.78–2.58)] increased the risk of these symptoms. Similarly, knowing one hospitalized person [AOR = 1.74 (95% CI = 1.40–2.16)] or more than one hospitalized person [AOR = 2.54 (95% CI = 2.10–3.09)], significantly increased the risk of anxiety symptoms. Having one person die due to COVID-19 [AOR = 1.81 (95% CI = 1.45–2.26)] or more than one person die due to COVID-19 [AOR = 2.93 (95% CI = 2.38–3.60)] significantly increased the risk of anxiety symptoms. For depression symptoms, knowing one infected person [AOR = 1.30 (95% CI = 1.03–1.63)] or more than one infected person [AOR= 1.33 (95% CI = 1.11–1.59)] increased the risk of these symptoms. Similarly, knowing one hospitalized person [AOR = 1.28 (95% CI = 1.04–1.59)] or more than one hospitalized person [AOR = 1.72 (95% CI = 1.43–2.07)], significantly increased the risk of depression symptoms. Having one family member/friend die due to COVID-19 infection [AOR = 1.48 (95% CI = 1.19–1.83)] or more than one family member/friend die due to COVID-19 infection [AOR = 2.06 (95% CI = 1.68–2.53)] significantly increased the risk of depression symptoms. Symptoms of both depression and anxiety (i.e., psychological distress) statistically significantly increased with having one or more than one family member or friend infected, hospitalized, or die due to COVID-19 (Table 3). In the final approach, the entire study population of non-infected adult Americans was grouped into two categories based on whether or not they had a family member/friend infected, hospitalized, or die due to COVID-19 (no vs. yes and ‘no’ was the comparison group; Table 3). Compared to those who did not have a family member/friend infected, hospitalized, or die of COVID-19, those who had one or more known person who was affected with COVID-19 infection were statistically significantly more likely to have depression symptoms, anxiety symptoms, or symptoms of both anxiety and depression.

## 4. Discussion

By August 2022, more than 95 million Americans were infected and more than a million died due to COVID-19 infection [1,21]. In this national study, 54% of the participants reported having a family member/friend infected with COVID-19 and 48% knew a person who was hospitalized due to COVID-19. These findings indicate the rampant spread of the virus across communities nationwide and the impact of this pandemic on the entire population, including those not infected. For example, having at least one family member/friend infected, hospitalized, or die from COVID-19 was found to be associated with an increased risk of anxiety or depression symptoms or both anxiety and depression (psychological distress). Also, the relationship between having a family member/friend infected, hospitalized, or die from COVID-19 and psychological distress had a dose-response pattern; the higher the number of family members/friends affected by COVID-19, the greater the odds of having symptoms of psychological distress among non-infected adult Americans. Furthermore, the odds of psychological distress symptoms increased in a graded manner among study participants based on the severity of COVID-19-related outcomes among family members/friends (i.e., odds of psychological distress increased from infection to hospitalization to death of a family member/friend due to COVID-19).

Compared to those who did not know anyone who was infected, those who knew one or more infected persons were statistically significantly more likely to have symptoms of both depression and anxiety (25% vs. 37% vs. 39%). The fear of getting infected by someone in the social networks, having a family member/friend in quarantine, uncertainty about the outcome of infection among family members/friends, work-family disruption, and stress due to the illness of a member in the social network could have led to the higher risk of anxiety and depression symptoms among non-infected individuals [7,8,9,12,18,22,23,24]. Similarly, compared to those who did not have a family member/friend hospitalized due to COVID-19 infection, those who had one or more family members/friends hospitalized due to COVID-19 were more likely to have symptoms of both depression and anxiety (23% vs. 37% vs. 46%). Recent studies suggest that hospitalized COVID-19 patients and their relatives may have a nearly equal prevalence of psychological distress. Also, the rates of psychological distress were found to be higher in relatives of COVID-19 intensive care patients than relatives of intensive care patients who had other health problems [23,24,25,26,27]. There are a myriad of reasons postulated for psychological distress, such as caregiver burden, uncertainty about infection outcomes in family members, fear of death of a dear one, not being supported by healthcare facilities and providers, communication-related challenges with medical providers and other family members, lack of social connections and support, financial concerns, added household responsibilities, and inability to see/meet COVID-19 infected family members (especially, if they are in an intensive care unit) [23,24,25,26,27,28,29]. Unfortunately, healthcare workers who took care of COVID-19 patients have also been shown to suffer similar or nearly the same levels of psychological distress [10,11].

An additional key finding in this national study was that more than a third (36%) of the participants had a family member/friend die due to COVID-19 infection. Compared to those who did not have a family member/friend die due to COVID-19, those who had one or more than one person die were significantly more likely to have symptoms of both depression and anxiety (24% vs. 39% vs. 51%). Studies before the pandemic established the relationship between sudden deaths in the family, bereavement, prolonged grief, and multidimensional psychological distress [29,30,31,32]. The COVID-19 pandemic has created unprecedented circumstances with mass bereavement, inability to see loved ones before death, overwhelming stress and fear, disruption of rituals/traditions, unusual management of dead bodies and funerals (to contain infection), unexpected life changes or alterations in family responsibilities and caregiving, losing a head of the household or income earners, multiple infected members in the household, and lack of emotional and social support for families who have lost a family member due to COVID-19 infection [28,29,30,31,32,33]. Unsurprisingly, the highest odds of psychological distress were observed among those who lost more than one family member or friend due to COVID-19 [AOR = 3.22 (95% CI = 2.61–3.96)].

Findings from our study provide significant information about the mental status of the general population throughout the United States during the COVID-19 pandemic (especially of those who had family members/friends/relatives hospitalized or die due to COVID-19 infection). For example, a back-of-the-envelope calculation suggests that by August 2022, there were approximately 200+ million Americans without a confirmed COVID-19 infection [1,21]. Based on our national survey findings, approximately half of the non-infected Americans had a family member/friend infected or hospitalized due to COVID-19 (equating to roughly 100 million people) and another third lost a family member/friend due to COVID-19 infection (equating to roughly 70 million people). Considering our study results, even if a third of these people now have new onset of symptoms of anxiety or depression due to COVID-19-related hospitalizations and death in the social networks, the estimated number of additional Americans with these symptoms would easily exceed 25 million people (in addition to more than 50 million who had a diagnosable mental illness before the pandemic) [3,12,15,20]. Experts estimate that without urgent interventions, the burden of mental disorders, associated disability, and loss of productivity will continue to increase in the U.S. due to the pandemic, and for each additional person needing mental healthcare (e.g., had a family member die due to COVID-19), the total cost could run into trillions of dollars [3,12,19]. The results of our national assessment indicate an urgent need to prioritize the exploration and implementation of multipronged interventions for mental health promotion that are customized to families, close relatives, and friends of those individuals who were hospitalized for or died due to COVID-19.

The United States has suffered some of the worst outbreaks of COVID-19 infections during the pandemic and profound upheaval across the nation. In response, federal and statewide efforts are underway to increase awareness about mental illness and the importance of obtaining help for psychological stress (e.g., online resources from government agencies) [34,35]. Additional initiatives and interventions are being explored by regional professional organizations, community-based agencies, worksites, and schools [33,34,36,37]. Experts have also made recommendations on bereavement services, grief counseling, coping with stress during public health emergencies, trauma-informed approaches to public mental health promotion, and best practices for healthcare workers who deal with psychological distress among patients and their relatives [23,24,25,26,27,28,29,30,31,32,38,39,40]. However, there remains a lack of coordinated national programs across countries to provide direct consumer services to address mental health problems during the COVID-19 pandemic. The need for the development and provision of mental health care-related programs designed to engage large population groups from various settings continues to increase, in part as a result of the ongoing COVID-19 pandemic. Two major areas of research need greater resources and attention. First, future research should focus on the scalability, reach, and impact of existing initiatives and interventions to determine the extent to which they mitigate psychological distress among those who have been affected by COVID-19 directly and indirectly. Second, innovations using technology should be explored to provide a wider array of population mental health promotion services [7,9,18,37,38].

In light of the burden of mental health problems nationally and globally, public health practitioners need to address this issue at both the educational level and policy level [39,40,41,42,43,44,45]. The major challenges that can be addressed through public health education interventions are improving communication with health care providers, enhancing communication within the social networks of individuals affected by COVID-19, enhancing the comprehension of medical information, promoting health literacy, providing information on grief/bereavement counseling and related services, reducing stigma around mental health problems and help-seeking, promoting stress management strategies, advertising resources available to the general public, and sensitizing people on the risk factors for poor mental health [42,43,44,45]. For policy-level interventions, there is an urgent need to allocate greater funding for mental health promotion interventions and strengthening the physical infrastructure and social services. For example, public policy initiatives should help increase community capacity for COVID-19 prevention and mental health promotion, resources for community mental health surveillance, mental health promotion interventions in healthcare systems, mental health related training and professional development for community health workers, workplace incentives and practices for physical and mental health promotion, support for schools/colleges to provide healthy lifestyle interventions and mental health/case management services, suicide prevention and awareness campaigns, group and family based interventions (e.g., problem solving, coping, etc.), creating pipelines of linguistically and culturally competent mental healthcare providers, primary care and community-based screening services and gatekeeping efforts, web-based interventions to promote sleep hygiene and stress management, community based strategies to reduce substance use and promote healthier lifestyles (e.g., exercise), social services/benefits for those who are most at risk (e.g., those who lost an income earner), telehealth and technology investments, and assistance with the deployment of community-based activities and services (e.g., phone lines, support groups, social/mass media channels, counseling sessions, bereavement support, funeral services, etc.) [37,38,39,40,41,42,43,44,45,46,47].

Most of the existing studies before this national assessment had critical limitations [23,24,25,26,27,28,29,30,31,32,33]. First, most of the existing studies were from hospital or healthcare facility-based samples of family members of infected/hospitalized patients. Second, these studies were mostly regional with small sample sizes. Third, the majority of the studies were outside the United States and were not population-based random samples. Fourth, the studies did not delineate the number of family members/friends infected, hospitalized, or dead due to COVID-19 (we used a scale of 0, 1, or more than 1 to assess the graded impact on psychological distress). Finally, earlier studies did not assess the psychological impact of COVID-19 infections among non-infected family members or friends [23,24,25,26,27,28,29,30,31,32,33].

### Limitations and Future Directions

Despite our attempt to address the aforementioned limitations of previous studies, the results of our study are restricted by all the threats to validity and reliability inherent to survey study design (e.g., reliance on self-reported behaviors, recall bias in participants, and the inability to establish cause-and-effect relationships). Another threat to validity is the nature of the sample (e.g., limited to those with internet connection or understanding of online surveys). Since the beginning of the COVID-19 pandemic, the United States has experienced several very distinct waves of infection surge. It could be possible that with each wave the psychological status or morbidity and mortality in social networks could have changed. We could not correlate the impact of such waves on psychological symptoms and COVID-19-related morbidity and mortality in social networks due to the cross-sectional nature of this one-time survey study. Also, we did not ascertain nuances such as the time since the death of a family member/friend or the duration of hospitalization of a family member/friend due to COVID-19; these details could have an impact on the level of psychological distress (i.e., temporal sequence of events and duration of distress).

Additional and longitudinal studies with larger samples of those who have had family members or friends hospitalized or die due to COVID-19 infection are warranted to understand the true and long-term burden of grief, worries, other mental health problems (e.g., PTSD), and psychological distress among adults and children affected. In addition, among those who had a family member or friend directly affected by COVID-19, future studies should also examine in community settings any confounders such as pre-existing mental illness, family structure, household wealth and resources, access to and usage of mental healthcare, social support, and relationship with those who were hospitalized or died due to COVID-19 [19,23,24,25,26]. An examination of such variables would provide precise and critical insights into the nature and extent of psychological problems among those who had a family member or friend infected with COVID-19. Finally, in addition to the aforementioned education and policy interventions, professional organizations and government agencies should increase funding for mental health research. As COVID-19 continues to disrupt the social and economic fabric of societies along with impacting the physical and mental health of the public, such funding is much needed and will be critical for our understanding of COVID-19-related fear, trauma, neurobiological and cognitive changes, and resilience. Such research initiatives will help design evidence-based interventions for rehabilitation, healing, and providing solutions for population mental health problems that have become rampant due to the ongoing COVID-19 assault [46,47,48].

## 5. Conclusions

In this national assessment, a large proportion of American people without a history of COVID-19 infection reported having a family member or friend infected, hospitalized, or die due to COVID-19 infection. These individuals were significantly more likely to have symptoms of psychological distress compared to those who did not have family members or friends infected with COVID-19. Family members, friends, and relatives of those affected by COVID-19 through infection, hospitalization, or death suffer from a higher level of anxiety and depression symptoms. There is an urgent need to develop educational interventions and implement policy measures that address the growing mental health needs and provide the necessary support to, this subgroup of the population not infected but indirectly affected by COVID-19 infections.

## Figures and Tables

**Table 1:** Background Characteristics of Study Participants Stratified by Anxiety and Depression Symptoms.

	Total	Anxiety Symptoms	Depression Symptoms	Psychological Distress
**Variable**	**N (%)**	**N (%)**	**N (%)**	**N (%)**
**All Participants**	2797 (100)	928 (33)	1198 (43)	899 (32)
**Sex**				
Male	1688 (60)	573 (34)	733 (43)	557 (33)
Female	1109 (40)	355 (32)	465 (42)	342 (31)
**Age Group**				
18–25 years	420 (15)	153 (36) *	200 (48) *	155 (37) *
26–35 years	1416 (50)	470 (33)	611 (43)	456 (32)
36–45 years	604 (22)	194 (32)	260 (43)	185 (31)
46–59 years	245 (9)	86 (35)	97 (40)	77 (31)
≥60 years	112 (4)	25 (22)	30 (27)	26 (23)
**Marital Status**				
Single/never married	791 (28)	269 (34)	330 (42)	264 (33) *
Married	1650 (59)	561 (34)	726 (44)	545 (33)
Engaged/living with a partner	184 (7)	54 (29)	74 (40)	52 (28)
Divorced/separated	133 (5)	32 (24)	52 (39)	26 (20)
Other (e.g., widow)	39 (2)	12 (31)	16 (41)	12 (31)
**Children at home**				
Yes	1649 (59)	600 (36) *	769 (47) *	582 (35) *
No	1148 (41)	328 (29)	429 (37)	317 (28)
**Race/Ethnicity**				
White	1591 (57)	606 (38) *	728 (46)	603 (38) *
Black	467 (17)	134 (29)	186 (40)	129 (28)
Asian	202 (7)	49 (24)	58 (29)	44 (22)
Multiracial	252 (9)	37 (15)	99 (39)	31 (12)
Other race	285 (10)	102 (36)	127 (45)	92 (32)
Hispanic	1141 (41)	418 (37)	617 (54) *	409 (36)
**Education**				
≤High school	282 (10)	95 (34)	103 (37)	92 (33)
Some college	800 (29)	228 (29)	303 (38)	209 (26)
Bachelor’s degree	1332 (48)	476 (36) *	624 (47) *	465 (35)
≥Master’s degree	383 (13)	129 (34)	168 (44)	133 (35) *
**Employment Status**				
Full-time	2137 (76)	736 (34) *	922 (43)	724 (34) *
Part-time	477 (17)	140 (29)	215 (45) *	132 (28)
Not employed	183 (7)	52 (28)	61 (33)	43 (24)
**Annual Household Income**				
0–$60,000	1555 (56)	597 (38) *	734 (47) *	574 (37) *
≥$60,001	1242 (44)	331 (27)	464 (37)	325 (26)
**Location**				
Rural	487 (17)	188 (39) *	237 (49) *	174 (36) *
Urban	1608 (58)	554 (35)	732 (46)	541 (34)
Suburban	702 (25)	186 (27)	229 (33)	184 (26)

* Indicates *p* < 0.05. N(%) Indicates frequencies and percentages. Psychological distress indicates symptoms of both depression and anxiety.

**Table 2:** COVID-19 Infection Among Family/Friends Stratified by Anxiety and Depression Symptoms.

	Total	Anxiety Symptoms	Depression Symptoms	Psychological Distress
**Variable**	**N (%)**	**N (%)**	**N (%)**	**N (%)**
**Family Members/Friends Infected with COVID-19**				
No	1284 (46)	333 (26)	525 (41)	321 (25)
Yes, one person	460 (16)	173 (38)	211 (46)	170 (37)
Yes, more than one person	1053 (38)	422 (40) *	462 (44)	408 (39) *
**Family Members/Friends Hospitalized due to COVID-19**				
No	1455 (52)	363 (25)	555 (38)	337 (23)
Yes, one person	561 (20)	211 (38)	250 (45)	206 (37)
Yes, more than one person	781 (28)	354 (45) *	393 (50) *	356 (46) *
**Family Members/Friends Died due to COVID-19**				
No	1773 (64)	460 (26)	670 (38)	433 (24)
Yes, one person	485 (17)	193 (40)	230 (47)	190 (39)
Yes, more than one person	539 (19)	275 (51) *	298 (55) *	276 (51) *

* Indicates *p* < 0.05. N(%) Indicates frequencies and percentages. Psychological distress = symptoms of both depression and anxiety.

**Table 3:** Multiple Regression to Predict Depression/Anxiety Symptoms Based on COVID-19 Infection Among Family/Friends.

**Predictor (3 Categories)**	**Anxiety** **Symptoms**	**Depression** **Symptoms**	**Psychological** **Distress**
	**AOR (95% CI)**	**AOR (95% CI)**	**AOR (95% CI)**
**Family Members/Friends Infected with COVID-19**			
No	Ref	Ref	Ref
Yes, one person	1.70 (1.34–2.15) *	1.30 (1.03–1.63) *	1.73 (1.36–2.21) *
Yes, more than one person	2.14 (1.78–2.58) **	1.33 (1.11–1.59) *	2.12 (1.75–2.56) **
**Family Members/Friends Hospitalized due to COVID-19**			
No	Ref	Ref	Ref
Yes, one person	1.74 (1.40–2.16) *	1.28 (1.04–1.59) *	1.85 (1.48–2.31) *
Yes, more than one person	2.54 (2.10–3.09) **	1.72 (1.43–2.07) *	2.84 (2.34–3.45) **
**Family Members/Friends Died due to COVID-19**			
No	Ref	Ref	Ref
Yes, one person	1.81 (1.45–2.26) *	1.48 (1.19–1.83) *	1.93 (1.54–2.40) *
Yes, more than one person	2.93 (2.38–3.60) **	2.06 (1.68–2.53) **	3.22 (2.61–3.96) **
**Predictor (2 Categories = No & Yes)**	**Anxiety** **Symptoms**	**Depression** **Symptoms**	**Psychological** **Distress**
	**AOR (95% CI)**	**AOR (95% CI)**	**AOR (95% CI)**
**Family Members/Friends Infected with COVID-19**			
No	Ref	Ref	Ref
Yes, one or more than one	1.90 (1.60–2.25) **	1.28 (1.08–1.53) **	1.86 (1.54–2.25) **
**Family Members/Friends Hospitalized due to COVID-19**			
No	Ref	Ref	Ref
Yes, one or more than one	2.00 (1.77–2.07) **	1.44 (1.26–1.71) **	2.25 (1.90–2.70) **
**Family Members/Friends Died due to COVID-19**			
No	Ref	Ref	Ref
Yes, one or more than one	2.27 (1.90–2.71) **	1.76 (1.48–2.08) **	2.46 (2.07–2.93) **

* Indicates *p* < 0.05. ** Indicates *p* < 0.01. AOR = adjusted odds ratios. 95% CI = confidence intervals. The binary outcomes were depression, anxiety, and moderate to severe psychological distress (yes vs. no). The predictor variable was COVID-19 infection, hospitalization, or death among family and friends (No was comparison group; Ref. OR = 1). Multiple regression analyses show the odds of various psychological outcomes after adjusting for the demographic characteristics from Table 1.

## Data Availability

The data presented in this study are available on request from the corresponding author.
